# Vav1 is necessary for PU.1 mediated upmodulation of miR‐29b in acute myeloid leukaemia‐derived cells

**DOI:** 10.1111/jcmm.13594

**Published:** 2018-03-13

**Authors:** Federica Vezzali, Silvia Grassilli, Elisabetta Lambertini, Federica Brugnoli, Simone Patergnani, Ervin Nika, Roberta Piva, Paolo Pinton, Silvano Capitani, Valeria Bertagnolo

**Affiliations:** ^1^ Department of Morphology, Surgery and Experimental Medicine Section of Anatomy and Histology University of Ferrara Ferrara Italy; ^2^ Department of Biomedical and Specialty Surgical Sciences University of Ferrara Ferrara Italy; ^3^ Department of Morphology, Surgery and Experimental Medicine Section of Pathology, Oncology and Experimental Biology University of Ferrara Ferrara Italy; ^4^ Laboratory for Technologies of Advanced Therapies (LTTA) University of Ferrara Ferrara Italy

**Keywords:** acute myeloid leukaemia, miR‐29b, PU.1, Vav1

## Abstract

It has been recently demonstrated that high pre‐treatment levels of miR‐29b positively correlated with the response of patients with acute myeloid leukaemia (AML) to hypomethylating agents. Upmodulation of miR‐29b by restoring its transcriptional machinery appears indeed a tool to improve therapeutic response in AML. In cells from acute promyelocytic leukaemia (APL), miR‐29b is regulated by PU.1, in turn upmodulated by agonists currently used to treat APL. We explored here the ability of PU.1 to also regulate miR‐29b in non‐APL cells, in order to identify agonists that, upmodulating PU.1 may be beneficial in hypomethylating agents‐based therapies. We found that PU.1 may regulate miR‐29b in the non‐APL Kasumi‐1 cells, showing the *t*(8;21) chromosomal rearrangement, which is prevalent in AML and correlated with a relatively low survival. We demonstrated that the PU.1‐mediated contribution of the 2 miR‐29b precursors is cell‐related and almost completely dependent on adequate levels of Vav1. Nuclear PU.1/Vav1 association accompanies the transcription of miR‐29b but, at variance with the APL‐derived NB4 cells, in which the protein is required for the association of PU.1 with both miRNA promoters, Vav1 is part of molecular complexes to the PU.1 consensus site in Kasumi‐1. Our results add new information on the transcriptional machinery that regulates miR‐29b expression in AML‐derived cells and may help in identifying drugs useful in upmodulation of this miRNA in pre‐treatment of patients with non‐APL leukaemia who can take advantage from hypomethylating agent‐based therapies.

## 1 INTRODUCTION

The use of hypomethylating agents has been found in recent years to correlate with a significant improvement in overall survival of patients with acute myeloid leukaemia (AML). In addition, despite the yet limited understanding of their mechanisms of action, molecules involved in DNA methylation seem to be efficient in treating older AML patients not able to tolerate standard intensive chemotherapy.^1^, ^2^, ^3^

A number of miRNAs have been described to reduce aberrant DNA hypermethylation, including miR‐29b,^4^ which is generally down‐regulated in AML and whose restoration into AML cell lines or primary samples led to a dramatic reduction of tumorigenicity.^5^, ^6^, ^7^ High pre‐treatments levels of miR‐29b have been recently associated with longer survival in AML patients treated with conventional chemotherapy and to improved clinical response to DNA methyltransferases (DNMT) inhibitors,^8^, ^9^ suggesting that strategies aimed to increase this miRNA may be useful in therapeutic DNA hypomethylation of leukaemic blasts. As downmodulation of miR‐29b correlates with different pathologies, a number of delivery systems for exogenous miR‐29b have been generated.^10^, ^11^, ^12^, ^13^ Concerning AML, a transferrin‐conjugated nanoparticle method was developed to increase miR‐29b in AML blasts.^14^ However, despite its efficacy in *in vivo* AML models, this approach is not clinically available at present and activation of transcriptional expression may constitute an efficient therapeutic strategy for restoring the miR‐29b level in myeloid leukaemia.

The precursors of the miR‐29 family are transcribed in 2 clusters: a miR‐29a/b1 cluster located on chromosome 7 (7q32) and a miR‐29b2/c cluster located on chromosome 1 (1q32). The 2 distinct precursor sequences encoding for miR‐29b lead to identical mature products.^10^ Different binding sites for several transcriptional factors have been identified in the promoter of miR‐29a/b1 and miR‐29b2/c clusters in different tissues.^5^ In myeloid leukaemia cells, CEBPα was reported to only regulate the miR‐29a/b1 cluster, providing the rationale for miR‐29b suppression in AML patients with loss of chromosome 7q or deficiency of this transcription factor.^15^ The miR‐29b2/c locus was shown to be activated at transcriptional level by the master myeloid regulator PU.1 in cells from acute promyelocytic leukaemia (APL) treated with all‐*trans*‐retinoic acid (ATRA).^16^ PU.1 is generally deregulated in AML by mechanisms including interference with binding sites (including at its own promoter site) by PML‐RARA 17 or disruption of PU.1 transactivation activity by RUNX1‐ETO (AML1‐ETO).^18^ Even if a number of differentiating agonists may restore the levels of PU.1 in AML‐derived cells,^19^, ^20^ at present, only APL patients are treated and take advantage by the use of differentiating therapies.^1^, ^2^

A crucial element of PU.1 transcriptional activity results from its ability to interact with a number of different protein partners, whose identification may provide the starting point for the development of targeted therapies for the treatment of haematological malignancies.^21^ In APL‐derived cells treated with ATRA, the interaction of PU.1 with its recognition sites on the CD11b 22 and miR‐142‐3p 23 promoters is entirely dependent from adequate amount of Vav1, a multidomain protein involved at different levels in agonist‐induced differentiation of APL‐derived promyelocytes.^24^ In addition, Vav1 may regulate protein expression of ATRA‐treated APL‐derived cells by determining the nuclear amount of proteins implicated in mRNA production and stability.^25^

This work was first aimed to assess the role of PU.1 in regulating the expression of miR‐29b in non‐APL cells, in order to identify agonists that, through upmodulation of this transcription factor, could be beneficial in DNA hypomethylation‐based therapies. We used Kasumi‐1 cells, showing the *t*(8;21) chromosomal translocation, that represents the most common cytogenetic subtype of AML. In this cell line, the ectopic expression of PU.1 overcomes its functional block induced by AML1‐ETO, in turn involved in a regulatory circuit with miR‐29b1 that controls the leukaemic phenotype.^18^, ^26^ As we demonstrated that, in APL‐derived cell treated with ATRA, Vav1 is crucial for the interaction of PU.1 with its DNA consensus regions on miR‐142 promoter, the cooperation between the 2 proteins in modulating miR‐29b expression was investigated in both APL‐ and non‐APL–derived cells.

## 2 MATERIALS AND METHODS

All reagents were from Sigma Chemicals Co. (St Louis, MO, USA) unless otherwise indicated.

### 2.1 Cell culture and treatments

The human myeloid leukaemia Kasumi‐1 (*t*(8;21)) and the APL‐derived NB4 (t(15;17) cell lines (German Collection of Microorganisms and Cell Cultures, Braunschweig, Germany) were cultured in RPMI 1640 (Gibco Laboratories, Grand Island, NY, USA) supplemented with 10% foetal bovine serum (FBS; Biowest, Nuaillé, France) at 37°C in a humidified atmosphere containing 5% CO_2_ in air. The cell density was maintained between 5 × 10^5^/mL and 1.5 × 10^6^/mL. Cells were monthly tested for mycoplasma and other contaminations and quarterly subjected to cell identification using single nucleotide polymorphism (SNP) typing.

Kasumi‐1 and NB4 cells were treated with 1 μmol/L ATRA dissolved in ethanol. Kasumi‐1 cells were treated with 100 nmol/L phorbol 12‐myristate 13‐acetate (PMA) suspended in DMSO.

To establish the percentage of adherent cells, after removal of cells in suspension, the cells adhering to the flask were detached with a trypsin/EDTA solution (Gibco Laboratories). Both suspended and adherent cells were counted using a hemocytometer, and the level of adhesion was expressed as a percentage of adherent cells over the total number of cells.

### 2.2 Immunoprecipitation and Western blot analysis

Total lysates from both Kasumi‐1 and NB4 cells were obtained by adding Laemmli's SDS sample buffer to cells, after washing with cold PBS containing 1 mmol/L Na_3_VO_4,_.

Purification of nuclei from both NB4 and Kasumi‐1 cells was performed essentially as previously reported,^22^ with the only modification consisting in the use of a 20‐gauge needle for Kasumi‐1.

For immunoprecipitation experiments, nuclei from ATRA‐treated NB4 and PMA‐treated Kasumi‐1 were lysed, added of protease and phosphatase inhibitors, were incubated with antibodies directed against Vav1 or PU.1 (Santa Cruz Biotechnology, Santa Cruz, CA, USA), and immunoprecipitated with protein A‐Sepharose (Pharmacia, Uppsala, Sweden), essentially as previously reported.^22^

For Western blot analysis, total cell lysates and immunoprecipitates from nuclei were separated on 8.5% polyacrylamide denaturing gels and blotted to nitrocellulose membranes (GE Healthcare Life Science, Little Chalfont, UK). The membranes were then reacted with antibodies directed against PU.1 and Vav1 (Santa Cruz Biotechnology) and against β‐tubulin (Sigma), incubated with peroxidase‐conjugated secondary antibodies and revealed using the ECL system (PerkinElmer, Boston, MA, USA), as previously reported.^22^ The chemiluminescence‐derived bands were acquired with ImageQuant™ LAS 4000 biomolecular imager (GE Healthcare), and the densitometrical analysis was performed by means of Image Quant TL software (GE Healthcare).

### 2.3 RNA interference assays

Exponentially growing Kasumi‐1 and NB4 cells were transfected with a mixture of small interfering RNAs (siRNAs; Santa Cruz Biotechnology) targeting the mRNAs for PU.1 or Vav1, using a previously described electroporation procedure.^22^, ^27^ As a control for transfection efficiency, which was always higher than 60%, a non‐silencing fluorescein‐labelled duplex RNA (Qiagen S.p.A, Milan, Italy) was used. 5 hours after transfection, cells were treated with ATRA or PMA, incubated at 37°C in a 5% CO_2_ atmosphere and then subjected to immunochemical or immunocytochemical analysis, to miR‐29b evaluation and to quantitative chromatin immunoprecipitation experiments.

### 2.4 Quantitative Real‐time PCR assay (qRT‐PCR)

High‐quality small RNAs from Kasumi‐1 and NB4 cells were extracted using a miRNeasy Micro Kit (Qiagen) as previously reported.^23^ Briefly, 10 ng RNA was subjected to single‐stranded cDNA synthesis, and the obtained cDNAs were employed as templates for quantitative Real‐time PCR‐based miR‐29b expression measurements using TaqMan MicroRNA Assays (ID 000413; Life Technologies). Thermal cycling and fluorescence detection were performed according to the manufacturer's instructions, using a Bio‐Rad CFX96™ sequence detection system (Bio‐Rad Laboratories, Hercules, CA, USA), and the data were analysed using a dedicated software (Bio‐Rad Laboratories). miRNA expression levels were normalized to U6 snRNA (Life Technologies), and fold change was determined using the 2^−ΔΔCt^ method. Cycle threshold >35 was excluded. Control PCR samples were run without cDNA. All reactions were performed in triplicate, and the experiments were repeated 3 times.

### 2.5 Quantitative chromatin immunoprecipitation (Q‐ChIP) assay

Quantitative chromatin immunoprecipitation experiments were performed on untreated and treated Kasumi‐1 and NB4 cells using a ChIP assay kit (Upstate Biotechnology, Lake Placid, NY, USA) as previously reported.^23^, ^27^ Samples were subjected to immunoprecipitation at 4°C overnight with antibodies directed against PU.1 or Vav1 or with a non‐specific IgG, used as a negative control (Santa Cruz Biotechnology). Beads were then washed, protein/DNA complexes eluted and cross‐links reversed by heating samples at 65°C overnight. After protein digestion, DNA was recovered using a PCR purification kit (Promega, Madison, WI, USA) in 50 μL elution buffer.

Quantitative PCR of (i) a 170‐bp DNA fragment, encompassing the putative PU.1 binding site located at −330/−324 bp from the transcriptional start in the human primiR‐29a/b1 promoter on chromosome 7q32.3 and of (ii) a 181‐bp DNA fragment, encompassing the putative PU.1 binding site located in the proximal miR‐29b2/c promoter on chromosome 1q32.2, was performed in triplicate using an iTaq Universal SYBR green SuperMix on a Bio‐Rad CFX96™ Real‐time detection system (Bio‐Rad Laboratories). The primers used were as follows: (i) Fw: 5′‐GCAGAGGATTAGACAGAGGGTG‐3′, Rev: 5′‐CTGAGAAGTGAGCAGCAACC‐3′; (ii) Fw: 5′‐GTTCTTCCCTGGACTTCTCG‐3′, Rev: 5′‐AAGCTGGTTTCACATGGTGG‐3′. Input corresponding to 1% of the total sonicated DNA was used as a positive control. All experiments were performed in triplicate.

To verify the ChIP specificity, samples from PMA‐treated Kasumi‐1 were subjected to PCR using primers amplifying a 131‐bp DNA region flanking the PU.1 binding site in the human primiR‐29a/b1 promoter on chromosome 7 (Fw: 5′‐GCAGGTTTTCAGTTGGTGGTTT‐3′; Rev: 5′‐GCCGTGACAGTTCAGTAGGA ‐3′), which was not predicted to bind PU.1.

ChIP‐qPCR data are presented as relative to input signals and in comparison with the background signals (IgG). PCR products were separated on tris‐acetate 1% agarose gels, stained with ethidium bromide and visualized by UV light apparatus.

### 2.6 Immunocytochemical analysis

ATRA‐treated NB4 cells were placed onto round 12‐mm glass coverslips by means of cytocentrifugation (Cytospin 3; Shandon Scientific, Astmoor, UK), as previously reported.^28^ Untreated and ATRA‐treated Kasumi‐1 cells were placed on glass dishes coated with 100 μg/mL Poly‐L‐Lysine to promote attachment of cells. PMA‐treated Kasumi‐1 cells that acquired adhesion capability were allowed to grow directly on coverslips. Samples on glass dishes were fixed with freshly prepared 4% paraformaldehyde (10 minutes at room temperature), washed in PBS and reacted with the anti‐PU.1 and anti‐Vav1 antibodies (Santa Cruz Biotechnology) in a NET Gel solution then incubated with FITC‐ and/or TRITC‐conjugated secondary antibodies, following a previously reported procedure.^22^ After washes, samples were reacted with 0.5 mg/mL 40,6‐diamidino‐2‐phenylindole (DAPI), dried with ethanol and mounted in glycerol containing 1,4‐diazabicyclo [2.2.2] octane (DABCO). Fluorescent samples were observed with a Nikon Eclipse TE2000‐E microscope (Nikon, Florence, Italy), acquiring cell images by the ACT‐1 software for a DXM1200F digital camera (Nikon).

For confocal analysis, after the labelling with secondary antibodies, samples were washed and incubated with TO‐PRO^®^‐3 Stain (Thermo Fisher Scientific, Paisley, UK), dried with ethanol and mounted in glycerol containing DABCO. Images were obtained using an Axiovert 220M confocal microscope equipped with a 100× oil immersion Plan‐Neofluar objective (NA 1.3, from Carl Zeiss, Göttingen, Germany) and a CoolSnap HQ CCD camera.

### 2.7 Statistical analysis

Statistical analysis was performed using the 2‐tailed Student's *t* test for unpaired data with the GraphPad Prism 6.0 statistical package (GraphPad Software, San Diego, CA, USA). *P* values < .05 were considered statistically significant.

## 3 RESULTS

### 3.1 PMA, but not ATRA, induces a PU.1 dependent increase in miR‐29b levels in Kasumi‐1 cells

To assess if PU.1 modulates the expression of miR‐29b in non‐APL myeloid cells, Kasumi‐1 cells, bearing the *t*(8;21) chromosomal translocation, were treated with agonists known to upmodulate this transcription factor, stemming from the notion that, in this cell line, ectopic expression of PU.1 overcomes its functional block induced by AML1‐ETO.^18^

**Figure 1:**
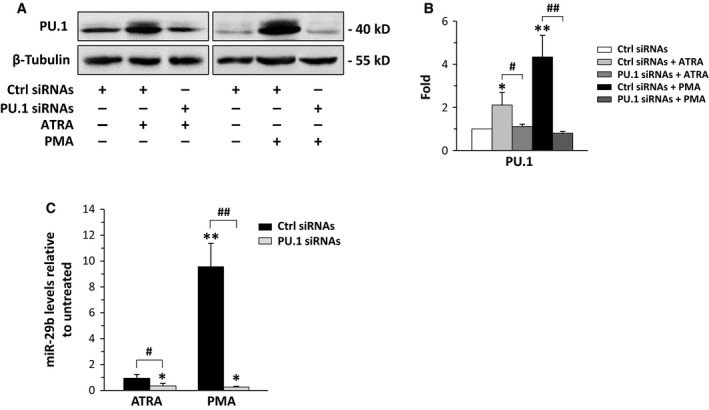
Modulation of PU.1 and miR‐29b levels in Kasumi‐1. (A) Representative Western blot analysis using the indicated antibodies of Kasumi‐1 cells in which PU.1 was down‐regulated during 72 h of all‐*trans*‐retinoic acid (ATRA) or phorbol 12‐myristate 13‐acetate (PMA) treatment. (B) Relative amounts of PU.1 as deduced from the densitometry of Western blot bands normalized with β‐Tubulin. Ctrl siRNAs: scramble siRNAs; PU.1 siRNAs: siRNAs specific for PU.1. The mean expression level of 3 separate experiments ±SD is shown. *P < .05, **P < .01 compared to respective controls (Ctrl siRNAs). (C) qRT‐PCR analysis of miR‐29b levels in Kasumi‐1 cells in which PU.1 was down‐regulated during treatment with ATRA or PMA. The values, obtained using the 2^−∆∆^ ^CT^ method, are shown as fold changes relative to the untreated condition and represent the means of 3 separate experiments ±SD. *P < .05, **P < .01 compared to untreated conditions. ^#^ *P* < .05, ^##^ *P* < .01

On the basis of the described PU.1‐mediated expression of miR‐29b induced by ATRA in NB4 cells,^16^ this agonist was first administered to Kasumi‐1. As expected,^29^ ATRA induced a slight cell adhesion (Figure S1A), indicative of a partial differentiation along the monocyte‐macrophage lineage, and a substantial increase in PU.1 (Figure 1A,B). Nevertheless, unlike what was observed in NB4 cells,^16^ ATRA failed to significantly upmodulates the miR‐29b levels in Kasumi‐1 (Figure 1C, Figure S1B).

We then treated Kasumi‐1 with PMA, known to activate PU.1 in myeloid cells.^30^ We found that PU.1 expression increased in treated conditions (Figure 1A,B), in parallel with the expected adhesion of this cell line (Figure S1C). At variance with ATRA, PMA induced a significant increase in miR‐29b expression (Figure 1C) that reached the maximum after 3 days of treatment (Figure S1D). Silencing PU.1 by specific siRNAs (Figure 1A,B) strongly counteracted the increase in miR‐29b induced by PMA (Figure 1C), indicative of the role of this transcription factor in mediating the agonist‐induced miRNA expression. On the other hand, the downmodulation of PU.1 also decreased the basal expression of miR‐29b in Kasumi‐1 cells, confirming its effective role in regulating the miRNA expression in this cell line (Figure 1C).

**Figure 2:**
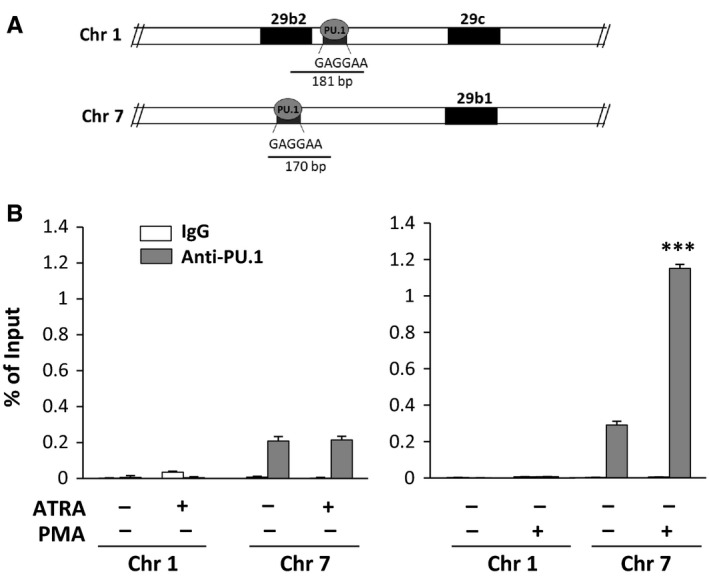
*In vivo* interaction of PU.1 with miR‐29b promoters. (A) Schematic representation of the putative PU.1 binding sites within the human miR‐29b2/c promoter on chromosome 1q32.2 and within the human miR‐29a/b1 promoter on chromosome 7q32.3. (B) Analysis of *in vivo* recruitment of PU.1 to both miR‐29b promoters performed by chromatin immunoprecipitation with an antibody directed against PU.1 in Kasumi‐1 cells treated with all‐*trans*‐retinoic acid (ATRA) or phorbol 12‐myristate 13‐acetate (PMA). The data are shown as percentage of the Input (genomic DNA collected before immunoprecipitation). IgG: negative control. Values represent the means of 3 separate experiments ±SD. ***P < .001 compared to untreated cells

As miR‐29b expression comes from the individual contribution of 2 different loci on chromosome 7q32.3 and on chromosome 1q32.2,^10^ the *in vivo* binding of PU.1 to miR‐29b promoters was investigated. For this purpose, ChIP assay was performed using specific primers that amplify the potential PU.1 binding sites in the 5′ regulatory regions (Figure 2A). As shown in Figure 2B, PU.1 was selectively recruited to the miR‐29b locus on chromosome 7 also in control conditions and PMA, but not ATRA, led to a significant increase in DNA associated with the transcription factor. A region of DNA flanking the miR‐29b promoter on Chr 7 and not predicted to bind PU.1 was also used in the ChIP assays, confirming the specific *in vivo* recruitment of this transcription factor in both control and PMA‐treated Kasumi‐1 (Figure S2)

### 3.2 In NB4 cells, Vav1 is essential for binding of PU.1 to its consensus sequences located on the miR‐29b promoters

**Figure 3:**
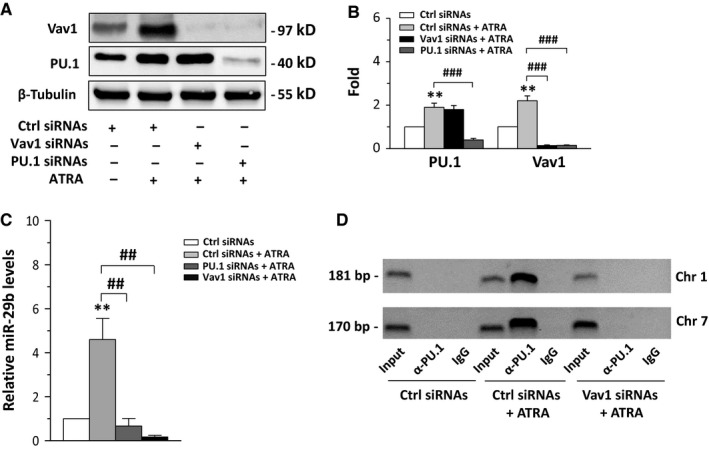
Vav1 and miR‐29b expressions in all‐*trans*‐retinoic acid (ATRA)‐treated NB4 cells. (A) Representative Western blot analysis, using the indicated antibodies, of NB4 cells in which PU.1 or Vav1 was downmodulated during 96 h of ATRA treatment. (B) Relative amounts of PU.1 and Vav1, as deduced from the densitometry of Western blot bands normalized with β‐Tubulin, used as internal control for equivalence of loaded proteins. The mean expression level of 3 separate experiments ±SD is shown. Ctrl: scramble siRNAs; PU.1 siRNAs: siRNAs specific for PU.1; Vav1 siRNAs: siRNAs specific for Vav1. (C) qRT‐PCR analysis of miR‐29b levels in NB4 cells in which PU.1 or Vav1 was down‐regulated during 96 h of ATRA treatment. The values are shown as fold changes relative to the untreated condition using the 2^−∆∆^ ^CT^ method and represent the means of 3 separate experiments ±SD. (D) Representative analysis of *in vivo* recruitment of PU.1 to human miR‐29b2/c and miR‐29a/b1 promoters by chromatin immunoprecipitation with an antibody directed against PU.1 in NB4 cells in which Vav1 was down‐regulated during ATRA treatment. The bands correspond to PCR products obtained amplifying a 181‐bp DNA fragment, encompassing the putative PU.1 binding site within the human miR‐29b2/c promoter on chromosome 1 and a 170‐bp DNA fragment encompassing the putative PU.1 binding site located in the proximal miR29a/b1 promoter on chromosome 7. Input: genomic DNA not subjected to immunoprecipitation (positive control); IgG: samples immunoprecipitated with a non‐specific antibody (negative control). **P < .01 compared to respective controls (Ctrl siRNAs) taken as 1. ^##^ *P* < .01, ^###^ *P* < .001

As we previously found that, in APL‐derived cells, Vav1 regulates the presence of PU.1 on its consensus region on the miR‐142‐3p promoter,^23^ our subsequent aim was to assess whether, also in regulation of miR‐29b, the PU.1 action is supported by Vav1. We first addressed this issue in NB4 cells treated with ATRA, in which, as expected,^22^ a significant increase in both PU.1 and Vav1 levels was induced by the agonist (Figure 3A,B). As expected,^16^ ATRA significantly upmodulated the expression of miR‐29b in this cell model (Figure 3C) that was completely abolished by silencing PU.1 or Vav1 with specific siRNAs (Figure 3A‐C).

ChIP assays performed with the anti‐PU.1 antibody did not reveal DNA associated with PU.1 in control conditions while ATRA induced the recruitment of PU.1 to the miR‐29b promoters on both chromosomes 1 and 7 (Figure 3D). Interestingly, when the cells were exposed to downmodulation of Vav1, the interaction of PU.1 with both miR‐29b promoters was abolished (Figure 3D), indicating that, also in this context, the ATRA‐mediated PU.1 recruitment to DNA required the presence of Vav1. ChIP experiments performed with an anti‐Vav1 antibody did not show significant amounts of DNA corresponding to the PU.1 consensus sequences on miR‐29b promoters in both untreated and ATRA‐treated NB4 cells (data not shown).

**Figure 4:**
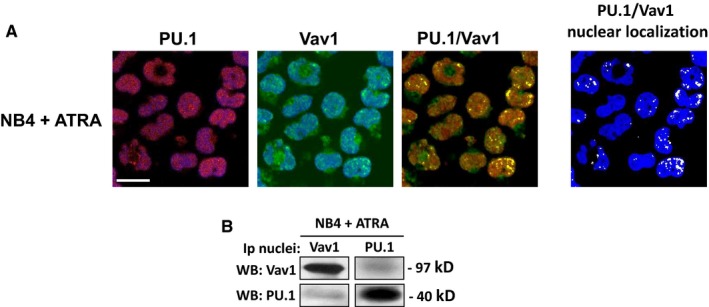
Vav1 and PU.1 association in all‐*trans*‐retinoic acid (ATRA)‐treated NB4 cells. (A) Representative confocal images of NB4 cells treated with ATRA and stained with antibodies against Vav1 (green staining) and PU.1 (red staining). TO‐PRO ^®^‐3 Stain was used to counterstain the nucleus (shown in blue). PU.1 and Vav1 images are shown as the overlay of the protein staining (red or green) with the staining of the nucleus (blue). Merged PU.1/Vav1 staining is shown with colocalization resulting in yellow. To the right, PU.1/Vav1 colocalization points were white coloured and overlapped to nuclear staining (blue). Bar = 20 μm. (B) Representative Western blot analysis with the indicated antibodies of Vav1 and PU.1 immunoprecipitates from nuclei of NB4 cells treated with ATRA. The data are representative of 3 separate experiments

The relationship between PU.1 and Vav1 investigated by confocal microscopy of ATRA‐treated NB4 cells stained with both anti‐PU.1 and anti‐Vav1 antibodies demonstrated an almost exclusive nuclear colocalization of the 2 molecules, within large speckled agglomerations (Figure 4A). Coimmunoprecipitation experiments with anti‐PU.1 and anti‐Vav1 antibodies confirmed our previous data 22 demonstrating the nuclear association of the 2 molecules in ATRA‐treated NB4 cells (Figure 4B).

### 3.3 In Kasumi‐1 cells, miR‐29b expression requires the presence of Vav1 in molecular complexes associated with the PU.1 binding site

**Figure 5:**
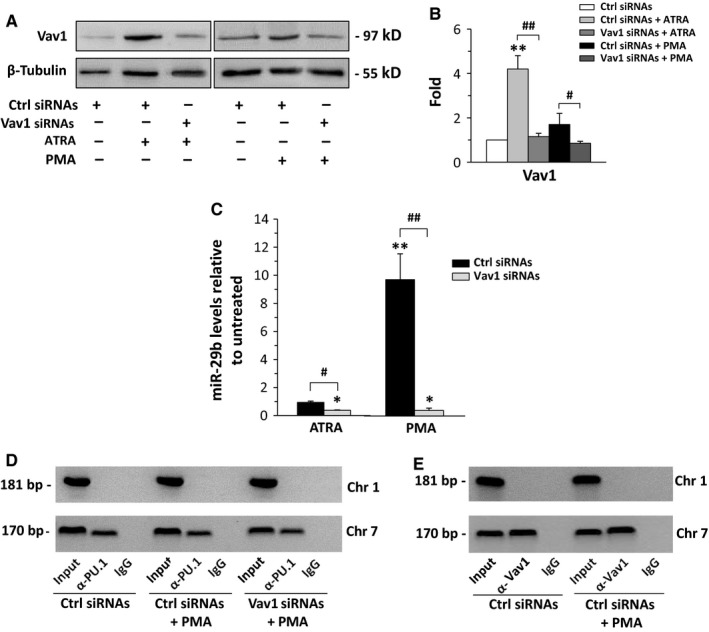
Vav1 and miR‐29b expression in Kasumi‐1 cells. (A) Representative Western blot analysis with the indicated antibodies of Kasumi‐1 cells in which Vav1 was down‐regulated during 72 h of all‐*trans*‐retinoic acid (ATRA) or phorbol 12‐myristate 13‐acetate (PMA) treatment. Ctrl: scramble siRNAs; Vav1 siRNAs: siRNAs specific for Vav1. (B) Relative amounts of Vav1 as deduced from the densitometry of Western blot bands normalized with β‐Tubulin, used as internal control for equivalence of loaded proteins. The mean expression level of 3 separate experiments ±SD is shown. **P < .01 compared to Ctrl siRNAs. ^#^ *P* < .05, ^##^ *P* < .01. (C) qRT‐PCR analysis of miR‐29b levels in Kasumi‐1 cells in which Vav1 was down‐regulated during ATRA or PMA treatment. The values are shown as fold changes relative to the untreated condition, determined with the 2^−∆∆^ ^CT^ method, and represent the means of 3 separate experiments ±SD. *P < .05 compared to untreated. ^#^ *P* < .05, ^##^ *P* < .01. (D) Representative analysis of *in vivo* recruitment of PU.1 to human miR‐29b2/c and miR‐29a/b1 promoters by chromatin immunoprecipitation in Kasumi‐1 cells in which Vav1 was down‐regulated during PMA treatment. The bands correspond to PCR products obtained amplifying a 181‐bp DNA fragment, encompassing the putative PU.1 binding site within the human miR‐29b2/c promoter on chromosome 1 and 170‐bp DNA fragment encompassing the putative PU.1 binding site located in the proximal miR29a/b1 promoter on chromosome 7. (E) Representative analysis of *in vivo* recruitment of Vav1 to human miR‐29b2/c and miR29a/b1 promoters by chromatin immunoprecipitation in Kasumi‐1 cells treated with PMA. Chromatin fragments were obtained by immunoprecipitation with an antibody directed against Vav1, and DNA was amplified by PCR. Input: genomic DNA not subjected to immunoprecipitation (positive control); IgG: samples immunoprecipitated with a non‐specific IgG (negative control)

On the basis of the results obtained with NB4 cells, the involvement of Vav1 in miR‐29b expression was investigated in Kasumi‐1 cells treated with ATRA or PMA. We revealed that, even if Vav1 is only upmodulated by ATRA treatment (Figure 5A,B), it silencing (Figure 5A,B) reduced the basal miR‐29b level in cells treated with ATRA and completely abrogated the increase in the miRNA induced by PMA (Figure 5C). This is indicative of the need of adequate Vav1 levels for the miR‐29b expression also in this cell line.

Unlike what we found in ATRA‐treated NB4 cells, the interaction of PU.1 with the miR‐29b promoter on chromosome 7 was not compromised in cells in which Vav1 was silenced during PMA treatment of Kasumi‐1 (Figure 5D). Interestingly, ChIP analysis performed with the anti‐Vav1 antibody and using primers able to amplify the PU.1 recognizing region within the miR‐29b promoters revealed significant amounts of DNA in Vav1 immunoprecipitates from both untreated and PMA‐treated Kasumi‐1 cells (Figure 5E). This suggests that, at variance with NB4 cells, the expression of miR‐29b in Kasumi‐1 requires the presence of Vav1 in molecular complexes on the PU.1 binding site on chromosome 7. The specificity of the ChIP assay was confirmed using a DNA flanking region not predicted to bind PU.1 (Figure S2).

**Figure 6:**
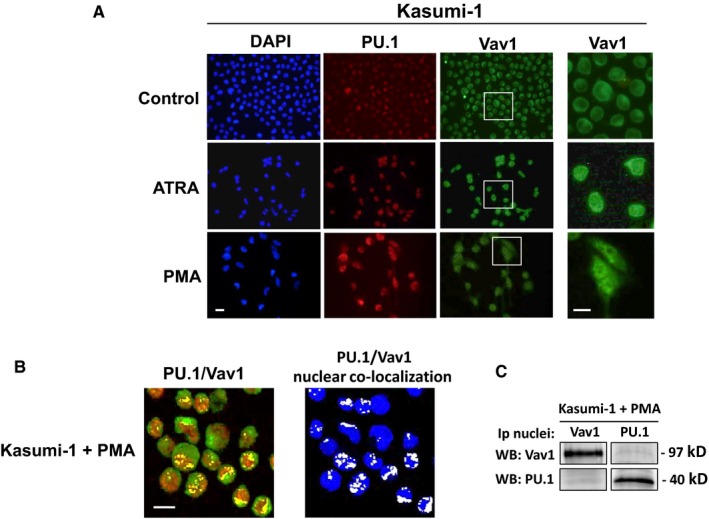
Vav1/PU.1 association in agonist‐treated Kasumi‐1 cells. (A) Representative fluorescence microscopy images of Kasumi‐1 cells treated with all‐*trans*‐retinoic acid (ATRA) or phorbol 12‐myristate 13‐acetate (PMA) for 3 days and subjected to immunocytochemical analysis with both anti‐PU.1 and anti‐Vav1 antibodies. Nuclei were stained with 4,6‐diamino‐2‐phenylindole (DAPI). A higher magnification of Vav1 stained cells is shown on the right. Bar = 20 μm. (B) Representative confocal immunofluorescence images of Kasumi‐1 cells treated with PMA and stained simultaneously with antibodies against Vav1 (green staining) and PU.1 (red staining). The nucleus was counterstained in blue using the TO‐PRO ^®^‐3 Stain. Merged staining of PU.1 and Vav1 is shown with colocalization resulting in yellow. To the right, PU.1/Vav1 colocalization points were white coloured and overlapped to the nuclear staining (blue). Bar = 20 μm. (C) Representative Western blot analysis with the indicated antibodies of Vav1 and PU.1 immunoprecipitates from nuclei of Kasumi‐1 cells treated with PMA

To assess whether the intracellular localization of Vav1 justifies the lack of effects of PU.1 induced by ATRA on miR‐29b expression in Kasumi‐1, immunocytochemical analysis of untreated and agonist‐treated cell was performed. As reported in Figure 6A, the untreated population comprises cells expressing different levels of PU.1, mainly located inside the nuclear compartment and upmodulated by both ATRA and PMA. Concerning Vav1, a cytoplasmic localization of the protein, describing the nuclear periphery, was observed in both untreated and ATRA‐treated cells (Figure 6A). At variance, PMA induced the accumulation of Vav1 inside the nuclear compartment, in which it showed a dotted staining (Figure 6A).

Confocal immunofluorescence analysis performed on Kasumi‐1 treated with PMA and simultaneously reacted with anti‐PU.1 and anti‐Vav1 antibodies revealed a large number of PU.1/Vav1 colocalization spots inside the nuclear compartment (Figure 6B). Coimmunoprecipitation experiments performed with anti‐PU.1 and anti‐Vav1 antibodies in nuclei from PMA‐treated Kasumi‐1 demonstrated, also in this cell model, the nuclear association between the 2 proteins (Figure 6C).

## 4 DISCUSSION

AML is predominantly a disease of older adults associated, also in case of karyotypes related to a favourable prognosis,^1^, ^2^ with poor long‐term outcomes with available therapies. In the last years, hypomethylating agents, traditionally employed in treatment of myelodysplastic syndrome, have shown efficacy in elderly patients with non‐APL myeloid leukaemia compared to that observed after intensive therapies.^3^ The level of miR‐29b seems to be important in response to hypomethylating agents, suggesting that strategies designed to increase this miRNA could effectively improve the prognosis of AML patients. At present, the absence of clinical application of delivery systems developed for synthetic miR‐29b in animal models 14 makes the activation of its transcription the only efficient strategy for restoring the miRNA level in myeloid leukaemia patients. As transcription factors responsible for miR‐29b expression are generally deregulated in AML,^5^, ^10^, ^17^ miR‐29b‐inducing compounds may be identified on the basis of their ability to restore the transcriptional machinery. As recent findings demonstrated that PU.1 is a transcriptional regulator of the miR‐29b2/c locus in APL‐derived cells,^16^ we investigated here the ability of this transcription factor to modulate miR‐29b in non‐APL cells, in order to identify agonists that, up‐regulating this transcription factor may be useful in hypomethylation‐based therapies. As experimental model, we choose the myeloid‐derived Kasumi‐1 cells, displaying the *t*(8;21) chromosomal rearrangement, the most common cytogenetic subtype of AML, whose survival rate is as low as 30% on a 5‐year basis.^3^ In Kasumi‐1, the notion that overexpression of PU.1 overcomes its functional block induced by the fusion protein AML1‐ETO, in turn involved in a phenotype regulatory circuit with miR‐29b,^26^ makes this cell line a suitable model to correlate the activity of PU.1 to the miRNA level. Despite the use of ATRA in non‐APL cells was ineffective in terms of differentiation and/or apoptosis,^31^ we first used this agonist to induce the expression of PU.1 in Kasumi‐1 cells, on the basis of its role on *miR‐29b* transcription in NB4 cells. Even though PU.1 increased, ATRA failed to induce the expression of miR‐29b in this cell model, indicating that the sole overexpression of PU.1 is not sufficient to activate the transcriptional machinery at the miR‐29b promoters. We then treated Kasumi‐1 with PMA, known to induce phosphorylation and nuclear translocation of PU.1 30 and to promote monocytic differentiation of this cell line,^32^ revealing that upmodulation of PU.1 is accompanied by a substantial increase in miR‐29b. The dramatic decrease in miR‐29b in both untreated and agonist‐treated Kasumi‐1 as a consequence of silencing of PU.1 allowed to unequivocally establish the involvement of this transcription factor in miR‐29b expression in this cell line.

To understand the rational of the agonist‐related effects of PU.1 on miR‐29b expression, we investigated the *in vivo* binding of PU.1 to both miRNA promoters in ATRA and PMA‐treated Kasumi‐1 cells. We revealed the association of PU.1 only with the miRNA promoter on chromosome 7, and we assessed that PMA, but not ATRA, increased the recruitment of the transcription factor to its consensus region at the miR‐29b1/c locus, leaving open the question of the ability of PU.1 to overcome the miRNA transcriptional block in this cell model.

Stemming from our previous data demonstrating that, in APL‐derived cells treated with ATRA, some PU.1 activities are correlated with the nuclear amount of Vav1, a multidomain protein variously involved in gene expression and mRNA processing,^24^, ^33^ we explored the PU.1/Vav1 relationship during expression of miR‐29b. We first investigated this issue in APL‐derived NB4 cells, in which we found that, at variance with Kasumi‐1, PU.1 is recruited at both miR‐29b2/c and miR‐29a/b1 loci as a consequence of ATRA administration. Similarly to what observed for regulation of CD11b and miR‐142‐3p,^22^, ^23^ we found that the interaction of PU.1 with both miR‐29b promoters in ATRA‐treated NB4 cells is entirely dependent from adequate levels of Vav1. In this cell model, Vav1 is not present in the PU.1‐containing molecular complexes on miR‐29b promoters but colocalizes with the transcription factor inside the nuclear compartment, confirming that a nuclear PU.1/Vav1 cooperation is necessary to regulate the agonist‐induced expression of miR‐29b in APL‐derived cells.

The same analysis performed in Kasumi‐1 cells revealed another scenario. In fact, we found that also in this cell model adequate levels of Vav1 are necessary for the expression of miR‐29b while, at variance with NB4 cells, this protein is not necessary for the interaction of PU.1 with the miRNA promoter. Interestingly, in Kasumi‐1, in contrast to NB4, we found the presence of Vav1 in the molecular complexes on the PU.1 consensus region on miR‐29b promoter, evidencing for the first time the direct participation of Vav1 to the transcriptional machinery in non‐APL cells.

Once we demonstrated the essential role of the cooperation between PU.1 and Vav1 in expression of miR‐29b in both NB4 and Kasumi‐1 cells, we tried to identify the events that prevented the induction of miR‐29b in ATRA‐treated Kasumi‐1, in which both PU.1 and Vav1 were upmodulated. The analysis of the intracellular distribution of the 2 proteins confirmed the nuclear localization of PU.1 in both ATRA and PMA‐treated cells, according to its transcriptional role. Concerning Vav1, its prevalent cytoplasmic localization was not substantially modified after ATRA treatment while this protein accumulated inside the nuclear compartment of Kasumi‐1 as a consequence of PMA treatment, similarly to what we have previously demonstrated in PMA‐treated NB4 cells.^28^ Inside the nucleus of PMA‐treated Kasumi‐1, PU.1/Vav1 association was revealed, justifying the presence of both molecules in protein/DNA complexes on the miR‐29b promoter and emphasizing their cooperation to other transcriptional activities. The lack of nuclear accumulation of Vav1 following treatment with ATRA could therefore be the basis of the inefficiency of this agonist on miR‐29b expression, suggesting that the only upmodulation of PU.1 is not sufficient and that the PU.1/Vav1 association inside the nuclear compartment seems to be a crucial event for inducing upmodulation of miR‐29b.

Our data allow to conclude that PU.1 may regulate the expression of miR‐29b in both APL and non‐APL cells and to establish that the contribution of the 2 miRNA precursors is cell‐related and almost completely dependent on Vav1 that plays peculiar roles in APL‐ and non‐APL–derived cell lines. Although not exhaustive, our results add new information about the transcriptional machinery that regulates miR‐29b expression in AML‐derived cells and may help in identifying drugs useful in pre‐treatments of non‐APL leukaemia, particularly of older patients, known to take advantage from therapies based on hypomethylating agents.

## CONFLICT OF INTEREST

The authors confirm that there are no conflict of interests.

## AUTHOR'S CONTRIBUTIONS

FV, SG, EL, FB and EN performed the research. SP and PP contributed essential tools. FV, EL and VB analysed the data. VB and FV designed the research study. FV, RP, SC and VB wrote the manuscript.
