# Supplementary figures and images for: Vav1 is necessary for PU.1 mediated upmodulation of miR‐29b in acute myeloid leukaemia‐derived cells
Source: J Cell Mol Med. 2018 Mar 13;22(6):3149–58. doi: 10.1111/jcmm.13594 (PMC5980196; doi:10.1111/jcmm.13594)

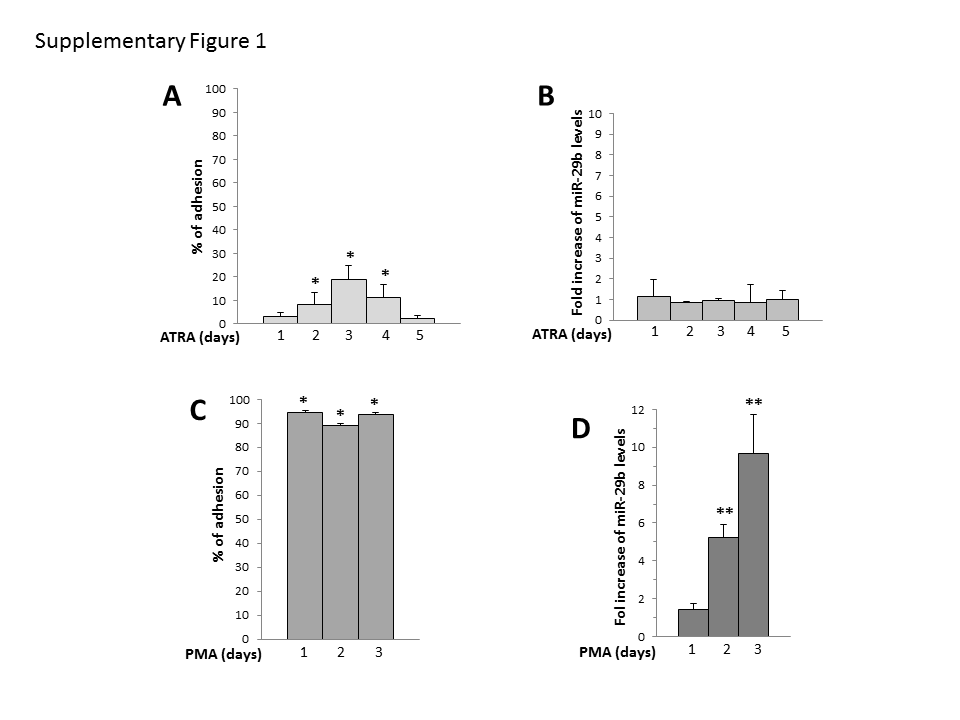

Supplement: Supplementary file 1 [file JCMM-22-3149-s001.TIF]

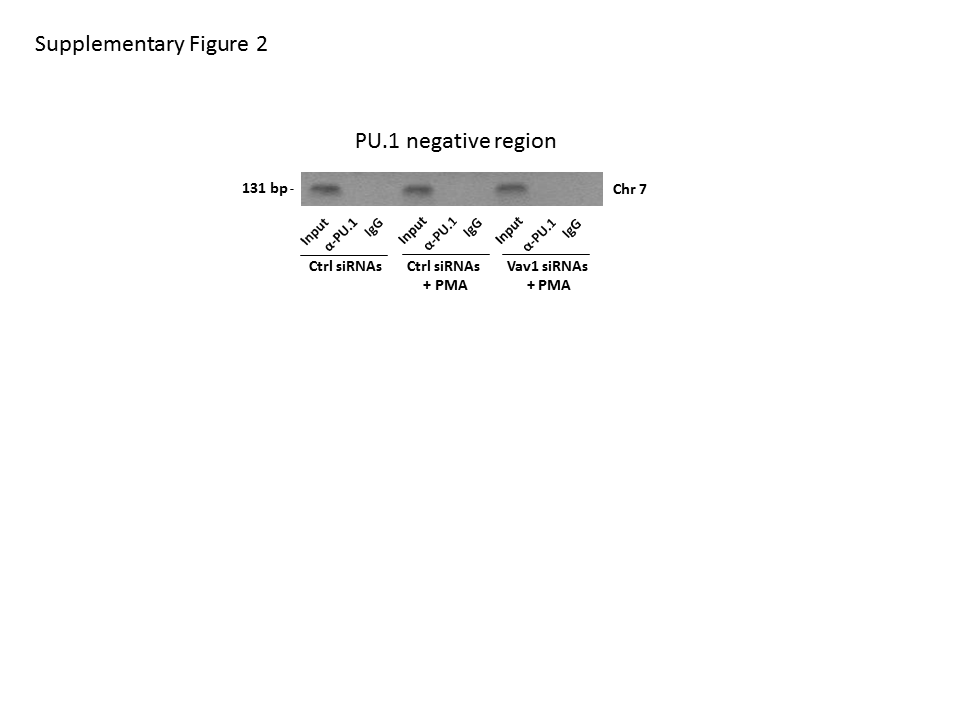

Supplement: Supplementary file 2 [file JCMM-22-3149-s002.TIF]
