# Supplementary material for: Vav1 is necessary for PU.1 mediated upmodulation of miR‐29b in acute myeloid leukaemia‐derived cells
Source: J Cell Mol Med. 2018 Mar 13;22(6):3149–58. doi: 10.1111/jcmm.13594 (PMC5980196; doi:10.1111/jcmm.13594)
Supplement: Supplementary file 3 [file JCMM-22-3149-s003.docx]

**Supplementary Figure 1** Adhesion and miR-29b levels in agonists-treated Kasumi-1 cells. Kasumi-1 cells treated with ATRA or PMA for the indicated time (Days) were evaluated for their adhesion capability (**A**, **C**) expressed as percentage of adherent cells over the total number of cells, and subjected to qRT-PCR analysis of miR-29b expression (**B**, **D**). MiR-29b levels are shown as fold changes relative to the untreated condition, by using the 2^-∆∆CT^ method, and represent the means of 3 separate experiments ± SD. *P <0.05, **P <0.01 compared to untreated.

**Supplementary Figure 2** ChIP assay on a PU.1 negative region. Representative analysis of chromatin immunoprecipitation with an antibody directed against PU.1 in Kasumi-1 cells in which Vav1 was down-regulated during PMA treatment. The bands correspond to the PCR products obtained amplifying a region of 131 bp containing a sequence located in the miR29a/b1 promoter on chromosome 7 not predicted to bind to PU.1. Ctrl siRNAs: scramble siRNAs; Vav1 siRNAs: siRNAs specific for Vav1. Input: genomic DNA not subjected to immunoprecipitation (positive control); IgG: samples immunoprecipitated with a non-specific IgG (negative control).
